# Growth and Temperature Properties of the Phase-Matching Angle of 23% Deuterated ADP Crystal

**DOI:** 10.3390/ma17020273

**Published:** 2024-01-05

**Authors:** Yuxiang Sun, Hongkai Ren, Shidong Zhuang, Xinle Wang

**Affiliations:** 1 School of Science, Shandong Jianzhu University, Jinan 250101, China; 11124@sdjzu.edu.cn; 2 Institute of Crystal Materials, Shandong University, Jinan 250100, China; renhk@sdu.edu.cn; 3 Department of Physics and Electronic Science, Weifang University, Weifang 261061, China; xinlewang@wfu.edu.cn

**Keywords:** DADP crystal, phase-matching angle, temperature property, spectral bandwidth, crystal growth

## Abstract

Theoretical analysis indicated that partially deuterated ammonium dihydrogen phosphate (DADP) crystal with a deuterium content of 23% could realize spectral noncritical phase-matching (S-NCPM) for type-I frequency doubling of an Nd:glass laser. To explore the temperature dependence of the phase matching (PM) angle of the second harmonic generation (SHG) process and the output SHG bandwidth of DADP crystal at 1.053 μm, we used the point-seed rapid-growth method to grow targeted DADP crystal with 23% deuterium content. Experimental results indicated that the grown DADP crystal had high quality and large dimensions (7 × 6 × 6 cm^3^). Using a femtosecond OPO laser as a tunable light source, the temperature dependence of the PM angle of the SHG process in DADP crystal at 1.053 μm was investigated. The PM angle changed linearly with temperature, as predicted by the theoretical calculation. In addition, under the condition of higher temperature, broad bandwidths of the second harmonic of DADP crystal were still observed. These results provide excellent guidance and reference value for the application of wavelength insensitive phase-matched second harmonic generation in partially deuterated DADP.

## 1. Introduction

Ammonium dihydrogen phosphate (NH_4_H_2_PO_4_, ADP) and its corresponding deuterated isomorph DADP are widely-known nonlinear optical crystals. Their crystal structures are similar to those of KDP crystal and belong to the point group $\bar{4}2$ m at room temperature. They can be applied for frequency conversion, such as the second and third harmonic generations [1,2], due to their large nonlinear optical (NLO) coefficient, high transmittance and high laser damage threshold (LDT) [3,4]. Compared with ADP crystal, DADP crystal can efficiently reduce stimulated Raman scattering (SRS) caused by a high-power laser [5,6]. Additionally, the deuterium of ADP crystal is able to increase the wavelength of the infrared cutoff edge and the transmittance in the near-infrared region [4]. In recent years, many efforts have succeeded in the frequency conversion and NLO properties of DADP crystals with different deuterium contents [7,8,9,10,11]. Our early studies of DADP crystal showed that for type-I SHG of different fundamental wavelengths in the range of 1.027~1.161 μm, DADP crystal can make first order wavelength sensitivity vanish via an adjustment in deuterium content [7]. These certain wavelengths are called retracting wavelengths or retracting points. Around these wavelengths, a change in incident laser wavelength does not significantly affect the conversion efficiency of a frequency-doubled laser. That is to say, when the center frequency of a broadband laser corresponds to the retracting point of a DADP crystal, the frequency components near the center frequency of the broadband laser can achieve both group velocity matching and high-efficiency harmonic conversion. This is important in broadband second harmonic conversion. In 2021, a large-aperture ADP crystal was cryo-cooled to reach non-critical phase matching for the fifth harmonic generation of a neodymium laser [8]. The deuteration of ADP crystal affected absorption peaks in the transmittance spectrum, UV absorption spectrum and Raman spectrum. The refractive indices of DADP crystals decreased with increasing wavelength and deuterated content [9]. Therefore, we could grow crystals with a specific retracting wavelength by adjusting the deuterium content in DADP crystals to achieve efficient frequency doubling at the target wavelength. Some work [9,10], as shown in Table 1, has been completed regarding narrowband second and third harmonic conversions at room temperature. However, little work has been undertaken regarding the temperature dependent PM angle and spectral characteristics of DADP crystal.

In this study, we selected more accurate data from DADP crystals to calculate the relationship equation between the retracing wavelength and the deuterium content of DADP crystals. Using this equation, we determined that a DADP crystal with a deuterium content of 23% could achieve wavelength insensitive SHG phase matching at 1053 nm. Next, we grew high-quality DADP crystal without visible macroscopic defects. Rocking curves showed excellent crystalline perfection and the Raman spectrum proved the accuracy of the deuterium content in the DADP crystal. When a broadband laser beam was incident on the DADP crystal, the PM angles of ADP and DADP crystals drifted linearly with the increase in temperature. At different temperatures, we measured and obtained frequency-doubled lasers with wide bandwidths. These results reveal that DADP crystals are excellent broadband frequency doubling crystals, and that both temperature and deuterium content are key factors determining the retracting points of DADP crystals.

## 2. Materials and Methods

### 2.1. Materials

#### 2.1.1. Retracing Curve

**Figure 1:**
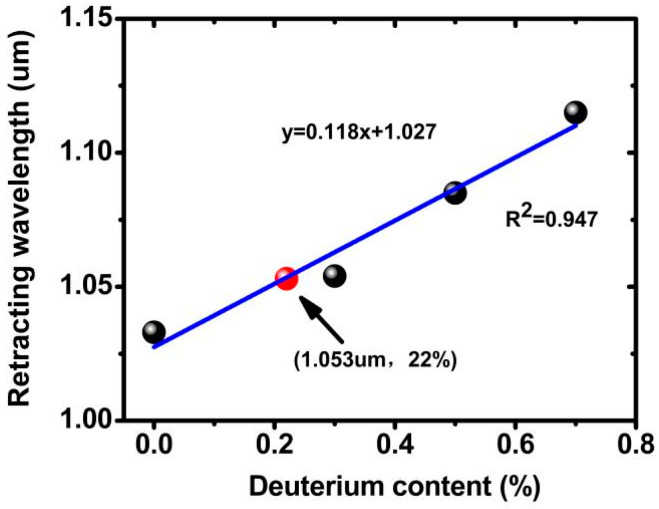
Calculated retracing wavelength as a function of deuteration level for partially deuterated ADP. Black bullets represented the retracting points calculated using the refractive index data of ADP crystals with different deuterations.

The retracing curve of homogeneous KDP crystals for SHG has been calculated by Mark S. Webb, indicating that deuterated ADP can shift the retracting point from 1.013 μm to 1.161 μm [11]. According to available data, we calculated retracing curves of ADP crystal from different refractive index values [12,13,14] and found that the positions of retracting points had an obvious discrepancy between them. The calculated values (at 1.030 μm) using Zernike and Zhu’s data had a high agreement. However, a considerable disagreement appeared when we calculated the same value (at 1.015 μm) using Kirby’s data, which had a deviation of about 0.015 μm. Of these results, the calculated value (at 1.030 μm) using Zhu’s data was selected to calculate the retracing curve and retracting points of partially deuterated ADP crystals. The calculated result is displayed in Figure 1. Based on crystal growth experience, the replacement of hydrogen atoms in crystals is linearly related to the deuterium content in the solution, so we chose to use linear fitting to fit these available data points.

From the above figure, the relationship equation between the retracing wavelength and the deuterium content of DADP crystal was

y = 0.118x + 1.027, (1)

where y was the retracing wavelength, and x was the deuteration level of DADP crystal. With the help of this equation, a (22 ± 0.5)% deuterated DADP could be acquired to grow a DADP crystal with a certain retracing wavelength at 1.053 μm. The corresponding value from Kirby’s data was 23.46%. So, we selected 23% deuteration as a target value to grow DADP crystal.

#### 2.1.2. Crystal Growth

**Figure 2:**
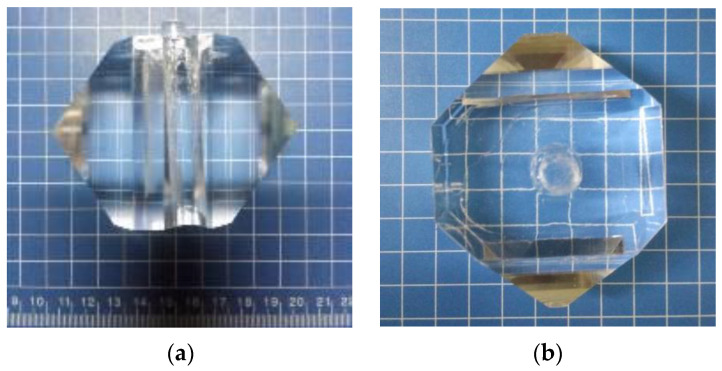
Photographs of (**a**) ADP and (**b**) DADP crystals grown using the point-seed technique.

ADP and DADP crystals were rapidly grown from pure and deuterated aqueous solutions, respectively, in standard 5000 mL glass crystallizers. For partially deuterated KDP or ADP crystals, it is well-known that deuterium contents in crystal and solution have significant discrepancies [15]. According to previous experience, the segregation coefficient in DADP crystal and its solution is ~0.83 under our growth conditions. So, to grow 23% deuterated ADP crystal, the deuterium content in the prepared solution was set to 27.7%. The pure aqueous solution was obtained by dissolving high-purity ADP raw material in deionized water according to a certain proportion. The deuterated aqueous solution was obtained by dissolving high-purity ADP raw material in heavy water by adding deionized water to control the degree of deuteration (23%). The solution was filtered using a polysulfone filter with a 0.22 µm pore diameter. According to the process of temperature reduction using the point-seed rapid-growth method at high supersaturation, ADP and DADP crystals started growing from a-cut point seeds. Our studies showed that, compared to the growth method using z-cut point seed, this method could obtain a high quality single crystal with excellent crystallization integrity and crystallization properties in a short period of time. Crystallization was performed in the temperature range of 5 °C, and the growth rate of the ADP and DADP crystals was about 3.5 mm d^−1^. The crystals were rotated in the ‘forward-stop-backward’ mode at a speed of 77 r min^−1^. All experiments were carried out in a thermostatic water bath, and the temperature was controlled using a programmable Shimada controller (Model FP21) (Hangzhou Bangshuo Electronics Co., Ltd., Hangzhou, China) with an accuracy of ±0.1 °C. There were no visible macroscopic defects in these crystals. Typical crystals are shown in Figure 2.

#### 2.1.3. Sample Preparation

**Figure 3:**
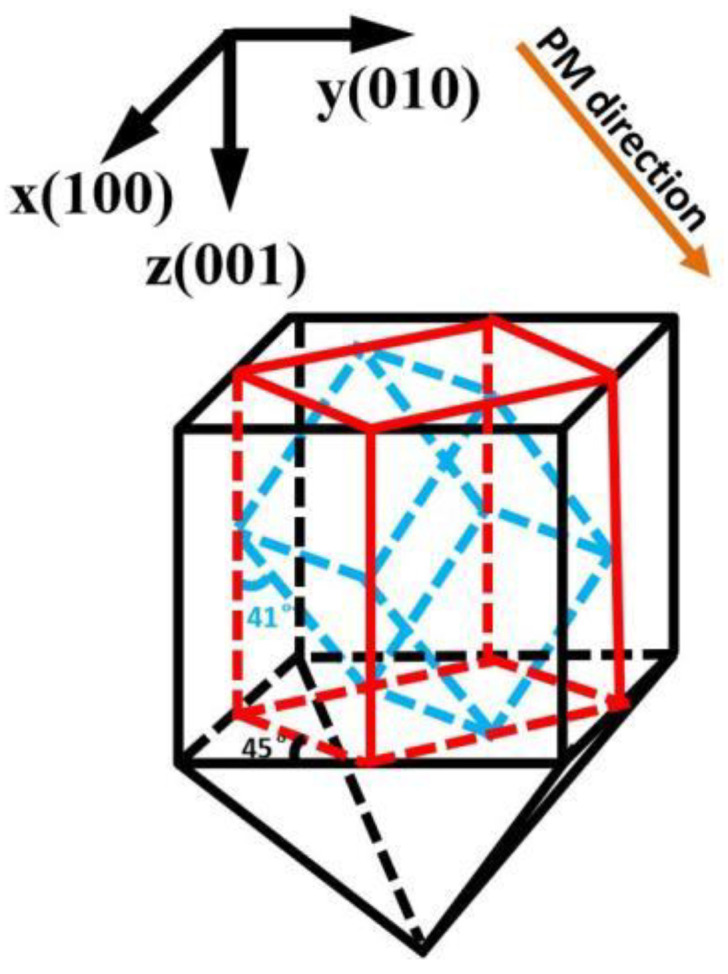
Crystal cutting. The red line represents the cutting direction of the first processing, and the blue line represents the cutting direction of the second processing.

The ADP and DADP samples’ dimensions in this study were all 10 × 10 × 10 mm^3^, and they were all cut along the type-I critical phase matching direction; that is, 45° in the X-axis direction (φ = 45°) and 41° in the Z-axis direction (θ = 41°). Their cutting accuracies were all less than 10′, and the transmittance faces of the crystals were polished, but not coated. The crystal-cutting process is shown in Figure 3. The red line in the figure represents the cutting direction of the first processing, and the blue line represents the cutting direction of the second processing. After these two steps were completed, transparent surfaces of the crystals along the PM direction were polished to obtain the samples we required.

### 2.2. Sample Characterizations

#### 2.2.1. Raman Spectroscopy

The deuteration level is an important parameter that significantly affects many essential properties of DADP crystals, such as the refractive index, electrooptical properties, thermal properties, electrical conduction and laser induced threshold [4,16,17]. In fact, the partition (segregation) coefficient between the crystal and the solution depends on the degree of supersaturation and temperature [18,19,20]. However, there are no reports about the deuterium partition coefficient of DADP crystals grown using the point-seed rapid method at high supersaturation. A novel experimental technique, based on the phase-matching angles measured at two wavelengths, has been used to determine the deuteration level of a DKDP crystal consistent with a specific index model [21]. However, this method still requires further validation. Raman spectroscopy is an effective way to determine deuteration levels in DADP crystals. The shift in the PO_4_^3−^ vibration peak can clearly be attributed to H/D isotope substitution. The observed main features are shifts in the PO_4_^3−^ vibration peak of the ADP crystal around 925 cm^−1^ and the shift toward a shorter wavenumber as deuteration levels increase [22]. In 1997, Yakshin et al. suggested that the Raman shift could be used to determine the deuteration degree of DKDP crystals [23].

**Figure 4:**
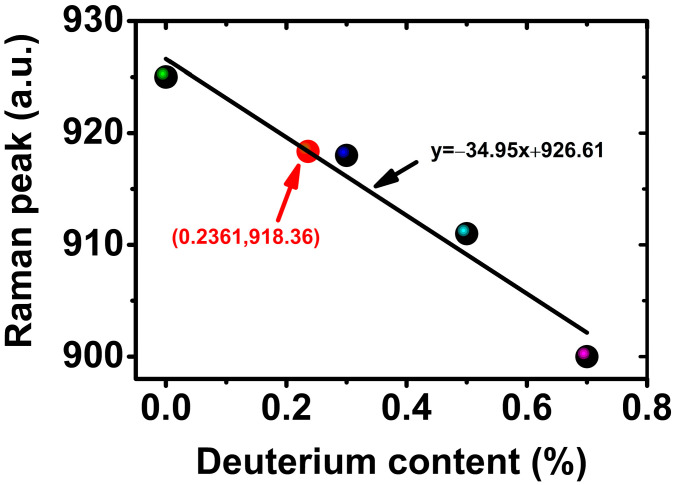
The relation curve of Raman peaks and corresponding deuteration levels of DADP crystals. Different colored bullets in the image represented the positions of Raman peaks of ADP crystals with different deuterations.

According to relevant research in this field, the characteristic peak at 925 cm^−1^ with a sharper shape was selected to confirm the deuteration level of DADP crystals. In order to match the retracing wavelength of homogeneous DADP crystals with the emission wavelength of the Nd:glass laser, we controlled the deuteration level of DADP crystals to adjust their retracing wavelength. A relation curve was fitted using a series of data from different DADP crystals [14] to determine the deuteration level of new DADP crystals; the fitted curve is shown in Figure 4.

By means of linear fit, the acquired plot equation was

y = −34.95x + 926.61,(2)

where y was the wavenumber of the PO_4_^3−^ vibration peak and x was the deuteration level of DADP crystal. The estimated deuteration level of DADP crystals was also about 23% according to the fitted curve.

**Figure 5:**
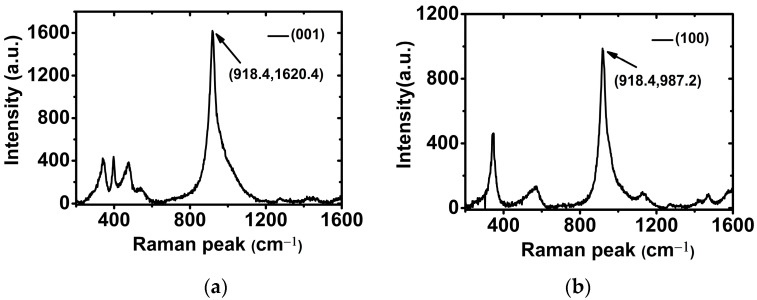
Raman spectra of DADP crystals (23%) at (**a**) (001) direction and (**b**) (100) direction.

Raman spectra of crystal samples were recorded in one step using a high-resolution (≤0.65 cm^−1^) confocal μ-Raman system (LabRAM HR800) with an excitation wavelength of 0.6328 μm and a scanning speed of 1 cm^−1^. The measured spectral range was from 200 to 1600 cm^−1^. In order to counter the influence caused by the H/D exchange process, the measuring point was taken inside each crystal sample on Z (001) and A (100), with the direction of the laser beam parallel to Z (001) and A (100), respectively. Raman spectra of DADP crystals (23%) are shown in Figure 5.

The positions of Raman peaks in both directions were all at (918.4 ± 0.2) cm^−1^, and an approximate 6.6 cm^−1^ shift appeared because of deuteration. This indicated that the position of Raman peaks had little relationship with the incident direction of the laser source, and that the grown DADP crystal had the expected deuterium content.

#### 2.2.2. Crystalline Perfection

**Figure 6:**
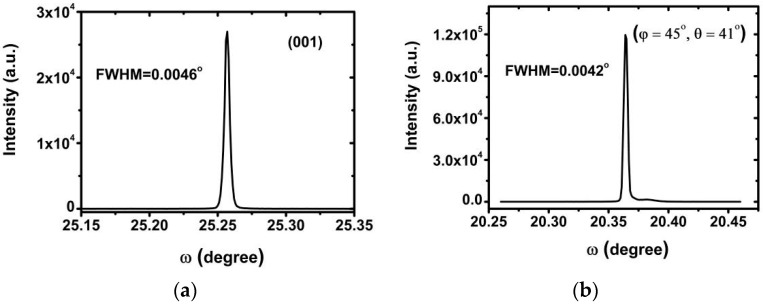
Rocking curves of (**a**) (001) and (**b**) ($45^{{}^{\circ}},41^{{}^{\circ}}$) faces of DADP crystal.

Crystalline perfection has a fundamental effect on the properties of crystals, and crystals with excellent crystalline perfection are the foundation of high optical qualities. A rocking curve can be used to describe the degree of angular divergence of a certain crystal face in a sample. The FWHM of a rocking curve, which presents information about the inner structure of crystals, can be measured to characterize the crystalline perfection of crystals [24]. A high resolution X-ray diffractometer (Bruker, D8 Discover, Bremen, Germany) was used in this study to measure rocking curves of different faces, as shown in Figure 6.

From the above curves, similar clear and sharp peaks with full widths at half maximums (FWHMs) of 0.0046° and 0.0042°, respectively, could be observed, which showed that the grown crystals had excellent crystalline perfection and high crystal quality. This was important to guarantee the samples’ high optical properties.

## 3. Experimental Set-Up

**Figure 7:**
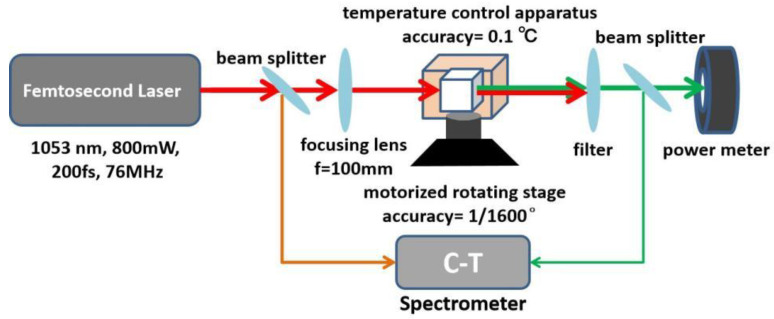
Experimental set-up.

The experimental setup used is shown in Figure 7. To provide a broadband laser light source, a femtosecond laser (COHERENT, Mira-OPO, Coherent Inc., Santa Clara, CA, USA, wavelength 1.053 μm, pulse width ~200 fs, repetition rate 76 MHz) with a bandwidth of about 0.012 μm was guided and focused, at normal incidence, into ADP and DADP crystals to achieve the second harmonic generation (SHG). The diameter of the spot where the laser exited was 3 mm. A beam splitter, collected using a CT spectrometer (IdeaOptics, FX4000, Shanghai Fuxiang Optics Co., Ltd., Shanghai, China), was mounted after the laser to separate the fundamental light to detect the wavelength and bandwidth of the fundamental light.

For enlarging the incident power density, a fused silica plano-convex lens (f = 100 mm) was used to focus the incident broadband laser. The focused points were all in the centers of crystals. The temperature of the ADP and DADP was controlled to within 0.1 °C by placing samples in a temperature-control apparatus for temperature-tuned phase matching. This temperature-control apparatus mainly consisted of a copper cube, a temperature sensor and a semiconductor heating piece. The copper cube was tightly sealed (one side was covered with optical glass and the other side was covered with quartz glass) to protect the ADP/DADP samples from air circulation and heat diffusion. A temperature sensor was located in the junction between the copper blocks and the crystal to accomplish temperature feedback and facilitate accurate control.

Crystals were heated via heat conduction between the copper blocks and the crystals. This energy was provided by a semiconductor heating piece placed between two copper blocks. The whole temperature-control apparatus was mounted on a 3D precise adjustment stage to adjust the optimal PM direction. A motorized rotating stage was adopted to rotate the crystals, and its angle sensitivity was up to 1/1600°. At each temperature, each crystal was rotated through a series of discrete angles, and conversion efficiencies were measured. The highest conversion efficiency corresponding to the PM angle at a given temperature was recorded. A filter was employed to separate the fundamental light and the second harmonic light because two lights existed simultaneously after the crystals. The second beam splitter, collected using the same spectrometer, was used to detect the wavelength and bandwidth of the second harmonic light. The residual second harmonic light was detected using a power meter. During the course of the whole experiment, spectra of fundamental light and second harmonic light were collected using the CT spectrometer for the sake of comparing their bandwidth discrepancies. Owing to the effects of the obvious discrepancy in deuterium content from the center to the boundary [25] and air flow on the temperature distribution in the temperature control scheme [26] of rapidly-grown crystal, the broadband beam was focused into the same point of the crystal for ten minutes to guarantee the equilibrium of this temperature-control apparatus throughout the whole experiment.

## 4. Results and Discussion

### 4.1. PM Angle Changes

**Figure 8:**
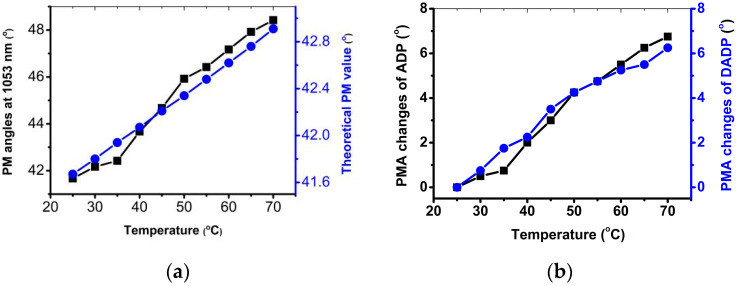
Comparisons of PMA variations with temperature at 1.053 μm (**a**) between actual and theoretical values of ADP crystal and (**b**) between ADP and DADP crystal.

The dependence of the PM angle on the temperature is one of the most important factors that result in phase mismatching. According to the available temperature-dependent Sellmeier equation [27], under the condition of an incident narrowband laser, PM angle variations with temperature at 1.053 μm of ADP crystal were calculated, and the result (blue curve) is shown in Figure 8a. As depicted in the figure, PM angle variations with temperature were extremely linear, and the PM angle of ADP crystal varied by an external angle of approximately 0.27° when the temperature deviated by 10 °C. The corresponding value for KDP crystal was 0.038° [28], which was much less than that of ADP crystal. It was deduced that the PM angle of ADP crystal was more sensitive to the crystal’s temperature.

When the incident laser became a broadband beam, a similar variation trend with a significantly larger variation scope appeared. In the black curve in Figure 8a, an average change of about 1.56° per 10 °C can be observed, and this variation was much larger than that of the previous condition. This indicated that the spatial bandwidth of the laser had an important influence on the PM angle, and that the PM angle under the condition of the broadband laser was more sensitive to the crystal’s temperature than it was in the case of a narrowband laser. DADP crystal’s PM angle variation is also displayed in Figure 8b. In Figure 8b, a similar variation trend can be observed, and its total variation range, which was about 6.5°, had a high agreement with that of ADP crystal. The change rate in the PM angle of DADP crystal with temperature was smaller than that of ADP crystal. These results indicated that the temperature dependence of the PM angle of DADP and ADP crystals were more sensitive to broadband laser.

### 4.2. Variation in Spectral Bandwidth before and after the Crystals

**Figure 9:**
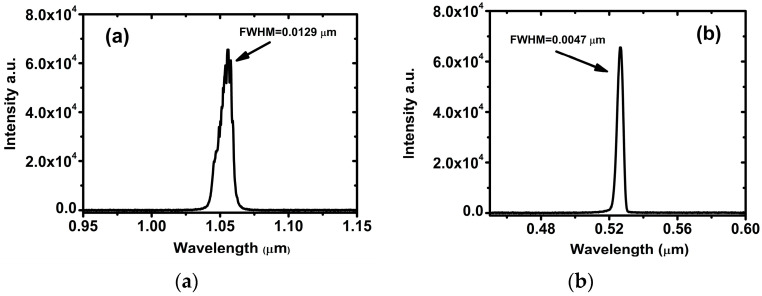
Spectra of (**a**) fundamental and (**b**) second harmonic waves of DADP crystal at room temperature.

In the process of second harmonic generation, owing to the spectral narrowing effect, the variation in spectral bandwidth has a vital impact on higher harmonic outputs [29]. A CT spectrometer was used to collect spectral bandwidth information before and after the crystals. Spectra of fundamental (1.053 μm) and second harmonic waves of DADP crystal at room temperature are displayed in Figure 9.

Through spectral data fitting of the fundamental and harmonic waves of DADP crystal, it was calculated that the bandwidths of fundamental and harmonic waves were 0.0129 μm and 0.00472 μm, respectively, at room temperature. At the same time, the corresponding spectral bandwidths of the fundamental and harmonic frequencies at 50 °C were 0.0143 μm and 0.00501 μm, respectively. These values at 70 °C were 0.0124 μm and 0.00475 μm, respectively. In comparison with the bandwidth of narrowband laser, both fundamental and harmonic waves had broader bandwidths. This difference was mainly caused by the bandwidth of the laser source. Comparing these results, we found that the influence of the incident laser bandwidth on the bandwidth of the frequency-doubled laser was much larger than that of the temperature was.

## 5. Conclusions

In this study, we grew DADP crystal with 23% deuterium content with the help of theoretical calculation. The grown crystal had excellent crystal quality. With an increase in temperature, DADP crystal showed a large range of linear shifts in PM angles, which was consistent with the theoretical calculation. When a broadband light source was incident on DADP crystal, we obtained a harmonic spectral bandwidth of approximately 0.005 μm. Experiments have shown that DADP crystal is an excellent broadband frequency doubling crystal. With changes in deuterium content and temperature, DADP can achieve broadband frequency doubling in a wide spectral range.

## Figures and Tables

**Table 1:** Nonlinear optical characteristics of ADP and DADP crystals.

Wavelength (μm)	Sample	α_0_ cm^−1^	β cm GW^−1^ (×10^−2^)	n_2_ cm^2^ W^−1^ (×10^−16^)	*Im*χ^3^ esu (×10^−16^)	*Re*χ^3^ esu (×10^−16^)	χ^3^ esu (×10^−16^)
1.064 [10]	ADP-I	0.78	0.23	-	0.68	-	-
	ADP-II	0.70	0.23	-	0.70	-	-
0.532 [10]	ADP-I	0.83	0.75	1.76	1.14	1.00	1.52
	ADP-II	0.73	0.92	1.34	1.40	0.77	1.60
0.355 [9,10]	ADP-I	0.99	0.78	2.01	0.82	1.18	1.44
	ADP-II	0.80	1.08	1.37	1.13	0.80	1.38
	30%DADP-I	-	8.01	48.8	0.086	0.030	0.091
	50%DADP-I	-	9.99	59.0	0.107	0.036	0.113

α_0_—the linear absorption coefficient, β—the nonlinear two-photon absorption coefficient, n_2_—the nonlinear refractive index, χ^3^—the third-order nonlinear susceptibility, *Im*χ^3^—the imaginary part of the third-order NLO susceptibility, *Re*χ^3^—the real part of the third-order NLO susceptibility.

## Data Availability

Data are contained within the article.
